# A Combined Computational and Experimental Approach to Studying Tropomyosin Kinase Receptor B Binders for Potential Treatment of Neurodegenerative Diseases

**DOI:** 10.3390/molecules29173992

**Published:** 2024-08-23

**Authors:** Duc D. Nguyen, Shomit Mansur, Lukasz Ciesla, Nora E. Gray, Shan Zhao, Yuping Bao

**Affiliations:** 1 Department of Mathematics, The University of Tennessee, Knoxville, TN 37996, USA; 2 Department of Chemical and Biological Engineering, The University of Alabama, Tuscaloosa, AL 35487, USA; smansur@crimson.ua.edu; 3 Department of Biological Sciences, The University of Alabama, Tuscaloosa, AL 35487, USA; lmciesla@ua.edu; 4 Department of Neurology, Oregon Health and Science University, Portland, OR 97239, USA; grayn@ohsu.edu; 5 Department of Mathematics, The University of Alabama, Tuscaloosa, AL 35487, USA; szhao@ua.edu

**Keywords:** tropomyosin kinase receptor B, drug discovery, TrkB docking, neurodegenerative disease

## Abstract

Tropomyosin kinase receptor B (TrkB) has been explored as a therapeutic target for neurological and psychiatric disorders. However, the development of TrkB agonists was hindered by our poor understanding of the TrkB agonist binding location and affinity (both affect the regulation of disorder types). This motivated us to develop a combined computational and experimental approach to study TrkB binders. First, we developed a docking method to simulate the binding affinity of TrkB and binders identified by our magnetic drug screening platform from Gotu kola extracts. The Fred Docking scores from the docking computation showed strong agreement with the experimental results. Subsequently, using this screening platform, we identified a list of compounds from the NIH clinical collection library and applied the same docking studies. From the Fred Docking scores, we selected two compounds for TrkB activation tests. Interestingly, the ability of the compounds to increase dendritic arborization in hippocampal neurons matched well with the computational results. Finally, we performed a detailed binding analysis of the top candidates and compared them with the best-characterized TrkB agonist, 7,8-dyhydroxyflavon. The screening platform directly identifies TrkB binders, and the computational approach allows for the quick selection of top candidates with potential biological activities based on the docking scores.

## 1. Introduction

Alzheimer’s disease (AD) has become one of the most challenging chronic age-associated diseases of the 21st century [1]. Despite years of intense research and numerous on-going trials, new therapies capable of delaying the onset, slowing progression, or improving the cognitive effects of AD are still needed [2,3]. Over 400 small molecular compounds developed in preclinical studies focusing on β-amyloid (Aβ) plaques or neurofibrillary tangles failed to progress through clinical trials [2]. The professionals in the field strongly suggest that alternative targets and approaches are explored to identify new potential drugs for the prevention and/or treatment of AD [4]. Numerous studies have shown the link between brain-derived neurotrophic factor/tropomyosin kinase receptor B (BDNF/TrkB) pathway activation and the improvement of neurological disorders, such as AD or major depressive disorder [5,6,7,8]. Accumulating evidence demonstrates BDNF, and its receptor TrkB, expression decrease in AD, and similar reductions exacerbate hippocampal dysfunction in animal models of AD [9]. Decreased levels of BDNF have also been reported in the serum and brain of AD patients [10,11,12]. Tau overexpression or hyperphosphorylation down-regulates BDNF expression in primary neurons and AD animal models [13,14,15]. Additionally, BDNF has been found to have protective effects on Aβ-induced neurotoxicity in vitro and in vivo [16], and BDNF administration directly into the rat brain has been shown to increase learning and memory in cognitively impaired animals [17]. Therefore, BDNF/TrkB signaling may serve as a valid target for ameliorating neurological and psychiatric disorders, including AD [8,18]. Targeting the BDNF/TrkB signaling pathway for the development of therapeutics for AD will potentially enhance our understanding of the disease [19]. Unfortunately, the natural ligand, BDNF, cannot penetrate the blood brain barrier (BBB), leading to poor bioavailability in the brain [20]. Alternatively, small molecule activators have been explored as a potential drug candidate targeting the BDNF/TrkB pathway [21,22,23], such as 7,8-dihydroxyflavone (7,8-DHF) [21,24,25], 7,8-DHF derivatives [26], and the mimetics of TrkB binding domains (loop II) of BDNF (e.g., LM22A compounds) [27,28]. For example, a derivative of 7,8-DHF (R13; 4-Oxo-2-phenyl-4H-chromene-7,8-diyl bis (methylcarbamate)) is currently under consideration as a potential drug for AD [26]. These studies suggest that small molecule agonists may have the potential as BDNF alternatives to activate the TrkB pathway. Recently, it has also been shown that several antidepressants (such as fluoxetine or imipramine) work through directly binding to TrkB and promoting BDNF signaling, further stressing the importance of pursuing TrkB as a valid target to treat various neurological disorders [29]. For example, TrkB activation in parvalbumin interneurons was required to promote reversal learning in spatial and fear memory by antidepressants [30]. However, the mechanism of small molecular TrkB activators has not been systematically studied in terms of binding location, the correlation of binding affinity of activities, and the structural diversity of small activators.

It has been a challenge to directly identify binders for transmembrane receptors because the receptors require boundary lipids for proper function. We recently developed a novel magnetic screening nanoplatform (MSN) based on cell-membrane-coated magnetic nanoclusters with immobilized receptors within the membrane [31,32]. The immobilized transmembrane receptors can be used to directly identify binding compounds through specific ligand–receptor interactions. This method works as a screening funnel to quickly narrow down a large number of molecules to binding compounds. The coated magnetic nanoclusters enable rapid binder isolation [32]. MSN can directly identify binders from a library mixture, and pure compounds are not necessary. In addition, effective approaches using transmembrane protein receptors as screening targets for direct compound identification are very limited [33,34,35] because transmembrane receptors require boundary lipids to function properly. MSN technology uses receptors within the cell membrane as functional receptors. The ability of MSN technology to screen compound mixtures and the use of transmembrane receptors as screening targets are two distinct benefits. The design of MSN technology was based on the central hypothesis that direct drug–receptor binding is essential to therapeutic functions. The screening experiments typically lead to a list of receptor binders. However, the binding affinity, biological activities, and differentiation between activators and inhibitors remain challenging. Taking TrkB as an example, several studies have shown multiple binding sites are available for this receptor. It is highly challenging to experimentally differentiate the binding location of a binder and highly labor- and cost-intensive to evaluate every single compound.

**Figure 1:**
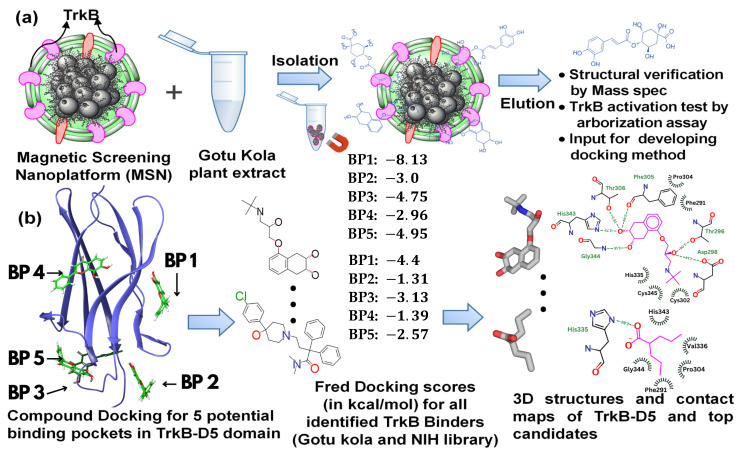
A schematic illustration of (**a**) the binder identification process using TrkB as the screening target, and (**b**) the docking computational studies and resulting information.

In this paper, we report a combined computational and experimental approach to studying TrkB binders for the potential treatment of neurodegenerative disease. Experimentally, we screened two different mixtures (Gotu Kola plant extract and the NIH clinical collection library [36]) using MSN screening technology with TrkB as the screening targets. The resulting TrkB binders were evaluated with Fred Docking software 2.2.0 [37], employing Chemgauss as its empirical-based scoring function, to elucidate the binding sites and binding affinity. Subsequent experiments were conducted to test the selected compounds. An overview of the experimental and computational process is illustrated in Figure 1. Our study suggested strong synergy between the docking results and compound activities evaluated by dendritic arborization in isolated Aβ-overexpressing hippocampal neurons. A strong association between TrkB activation and neuron development was identified. Furthermore, our findings demonstrate the feasibility of using docking studies to systematically explore all potential binding sites, allowing one to effectively narrow down the top candidate compounds. Our combined approach will not only greatly benefit drug discovery processes using TrkB transmembrane proteins as targets but also allow for the evaluation and validation of any previously reported TrkB binders.

## 2. Results

### 2.1. Magnetic Drug Screening Nanoplatform

Our recently developed MSN based on cell-membrane-coated magnetic nanoclusters with immobilized TrkB allowed for the direct identification of TrkB binders from mixtures. This screening platform is able to identify and extract binding compounds through specific ligand–receptor interactions, which quickly narrows down a large number of molecules to binding compounds, while magnetic iron oxide nanoclusters inside enable rapid binder isolation (Figure 1a). We demonstrated the proof-of-concept [31] and feasibility [32] of this MSN technology to identify binders from small-molecule libraries and plant extracts. Using this MSN screening platform with TrkB as the screening target, we have effectively identified a list of TrkB binders from Gotu Kola plant extract [32]. To evaluate whether the TrkB binders have the anticipated biological activity of activating TrkB, we performed preliminary testing on one of the TrkB binders, 4-O-cafeoylquinic acid (4-O-CQA), using dendritic arborization assay. The assay was performed using hippocampal neurons isolated from 5xFAD mice and cultured in vitro [32]. The isolated 5xFAD hippocampal neurons treated with 4-OCQA at 1 μM for several days significantly increased arborization in these Aβ-expressing neurons [32].

However, it is a highly labor-intensive and costly process to evaluate the biological activity of every identified TrkB binder. In addition, the screening process only leads to binders, but no quantitative affinity information and no receptor binding sites can be obtained. Therefore, we explored a docking computation approach to evaluate the binding affinity of the TrkB binders and to elucidate the binding location within the receptor.

### 2.2. Docking of TrkB Binding Compounds from Gotu Kola Plant Extract

**Figure 2:**
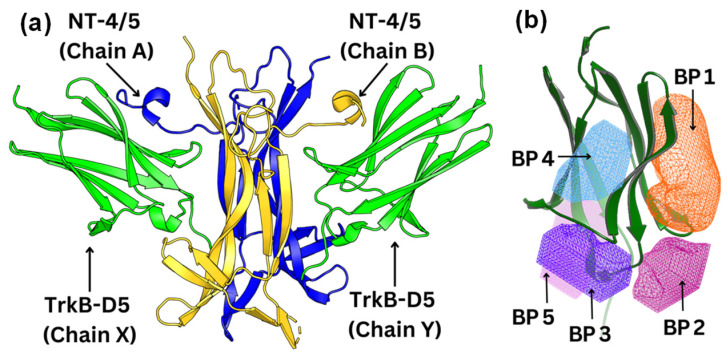
(**a**) Structure of the TrkB-D5:NT-4/5 complex (PDBID: 1HCF). TrkB-D5 is colored green (chains X and Y), and NT-4/5 homodimer is colored blue (chain A) and gold (chain B). (**b**) Five potential binding pockets in TrkB-D5 domain, where BP stands for Binding Pocket.

To visualize and identify the binding mechanism of the Gotu Kola plant compounds, we selected TrkB-D5 as the receptor for docking these ligands. TrkB-D5 is considered as a favorable target for neurological and psychiatric disorder agonists, which bind to this TrkB domain to mimic the binding of Brain-Derived Neurotrophic Factor (BDNF) [38,39,40,41,42]. We leveraged the high-resolution X-ray crystallographic data of TrkB-D5 in the NT-4/5TrkB complex (PDB ID: 1HCF) [43]. Derived from human sources and accessible through protein databanks [44], this complex comprises four chains designated as A, B, X, and Y. In this configuration, chains A and B form a neurotrophin 4 homodimer, while chains X and Y correspond to two monomers of TrkB-D5. Figure 2a depicts the structure of TrkB-D5:NT-4/5 complex formed by chain X and chain A. Our research primarily concentrates on chain X of PDB ID 1HCF to simulate the docking process between the TrkB-D5 domain and selected agonist candidates. The protocol for optimizing this structure is detailed in the Docking Computation section.

Using the OEDocking Graphical User Interface [37], we identified five potential binding regions for the TrkB agonists as depicted in Figure 2b. In our study, we utilized the FRED docking score with the Chemgauss4 scoring function [37], which enhances molecular docking accuracy by refining the assessment of hydrogen bond directionality and metal chelator interactions, to rank the most favorable binding pockets for our TrkB binders (Table 1). The default parameters in the FRED 2.2.0 software were employed in this study. In particular, while Chemgauss4 is used for the optimization phase, Chemgauss3 is utilized for the exhaustive search phase. Furthermore, the FRED software defines the “negative image” to eliminate poses that either clash with the protein or extend too far from the binding site. Specifically, the negative image describes the shape of the active site and is stored as a potential grid surrounding it. This image highlights areas where ligand atoms can make many contacts with the active site atoms without clashing and indicates likely positions for ligand atoms during optimal binding. According to the Fred Docking scores in Table 1, binding site 1 (BP 1) is the most favorable region for the investigated agonists, but depending on the compound, the other four binding sites are also involved to some extent. Table S2 presents a detailed analysis of the binding site regions (BP1 to BP5) within the TrkB-D5 protein, highlighting the key amino acids responsible for hydrogen bonding and hydrophobic interactions. Each binding site region is defined by a unique set of amino acids that contribute to the overall stability and function of the protein–ligand complex. Interestingly, our experimentally tested bioactive compound, 4-O-cafeoylquinic acid, showed high levels of binding affinity to all five binding sites and with much high binding in site 1. Although dicaffeopllauiric acid and castillicetin have slightly stronger binding to BP1 than 4-O-cafeoylquinic acid, the involvement of other binding sites is significantly lower than 4-O-cafeoylquinic acid. The trend is very similar to the Fred Docking scores of the known TrkB agonist, 7,8-dihydroxyflavone, where BP1 has high binding affinity, with the involvement of all other four binding sites. The synergy between the docking studies and the experimental observation suggests that our docking computation provides an effective tool to predict the activity of TrkB binders.

### 2.3. Screening of the NIH Clinical Collection Library

The plant extracts serve as a great resource for bioactive compounds; however, because of the large number of compounds and the diverse structures, the properties of certain compounds are not readily available. Therefore, we further applied our MSN technology to screen the NIH clinical collection library. The NIH clinical collection library contains 708 compounds used in human clinical trials that have known therapeutic functions, well understood molecular mechanisms of action, and available structural and physicochemical properties. Following a similar procedure to that used to screen Gotu kola plant extracts and to the compound analysis process, we have identified 17 compounds as TrkB specific binders. The binder identification process based on ultra-performance liquid chromatography (UPLC) chromatograms of binders eluted from MSN with TrkB and MSN without TrkB was shown in Figure S1. Peaks only appeared in UPLC chromatograms of MSN with functional TrkB but not in control MSN from TrkB-null cells were analyzed by mass spectrometry as binders. Mass spetrometry was performed in both negative and positive ionization mode, but the positive mode exhibits much better signals. Therefore, positive ionization mode was used to analyze the TrkB binders. The compound matching process was achieved by comprising the compound-detected masses with the NIH compound data (Figure S2). Subsequently, we applied the same docking studies described previously to the identified compounds.

The identified TrkB binders along with the Fred Docking scores (in kcal/mol) for all five different binding sites are shown in Table 2. Similar to the binding behaviors of the TrkB binders from Gotu kola plant extracts, BP1 is the favorable binding site for all the binders from the NIH library. In addition, the binding affinities for the stronger binders were also very close such as dicaffeopllauinic acid (−7.63 kcal/mol) and 4-O-cafeoylquinic acid (−7.02 kcal/mol) for binders from plant extracts and nadolol (−8.13 kcal/mol) and clofazimine (−7.36 kcal/mol) for binders from the NIH library.

**Figure 3:**
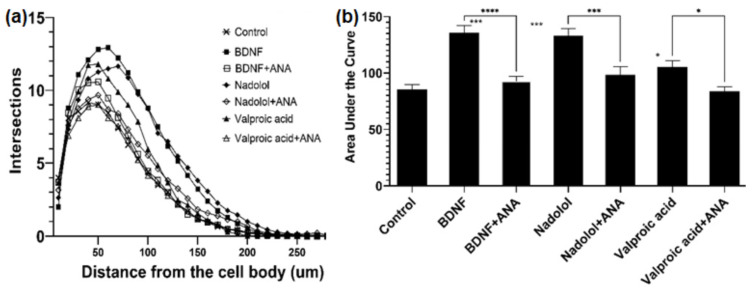
TrkB activating compounds increase dendritic arborization in hippocampal neurons. (**a**) Treatment with BDNF (25 ng/mL), nadolol (10 nM), and valproic acid (500 uM) increased dendritic arborization in primary hippocampal neurons isolated from embryonic C57BL6 mice as quantified by sholl analysis. The effects of BDNF, nadolol, and valproic acid were attenuated by co-treatment with the TrkB inhibitor ANA-12 (100 uM). (**b**) Area under the curves calculated from panel A * *p* < 0.05, *** *p* < 0.001, **** *p* < 0.0001.

Nadolol is currently an FDA-approved β-blocker with known therapeutic functions; well-understood molecular mechanisms of action; and available solubility, safety, and pharmacokinetic properties. Most importantly, β-blockers have been shown to lower the risks of AD [45]. In fact, the phase I trial is in process for studying the cognitive impacts of the combination of clenbuterol and nadolol on mild dementia due to AD or other diseases [46]. In contrast, valproic acid that has been commonly used in the treatment of epilepsy and bipolar disorder, was shown to be related to TrkB [47], and is considered to be a partial TrkB agonist. The ability of MSN with functional TrkB to identify valproic acid also indicated the effectiveness of MSN for TrkB binder identification. Therefore, those two compounds were selected as top candidates for TrkB activation associated with neuron elongation using primary hippocampal neurons isolated from embryonic C57BL6 mice. First, the isolated primary neurons were treated with BDNF, the physiological ligand in the human body. Primary neurons exposed to 25 ng/mL of BDNF were observed to have increased arborization, which is directly linked to increased neuroplasticity. The arborization was attenuated in the presence of the TrkB selective inhibitor, ANA-12, as shown in Figure 3. Similarly, we tested valproic acid and nadolol using the same dendritic arborization assay. Both molecules are FDA-approved drugs with well-established bioavailability, toxicity, and BBB penetration data. Specifically, valproic acid is readily soluble in water (50 mg/mL) and penetrates the BBB [48]. Nadolol is slightly soluble in water (46.4 µg/mL) and has been previously shown to cross the BBB [49]. Valproic acid at 500 µM and nadolol at 10 nM concentration increased dendritic arborization compared to the control. The increase in arborization elicited by nadolol was as effective as the one observed for BDNF, the positive control. The increase in dendritic arborization caused by valproic acid treatment was less effective than nadolol treatment but was significantly higher compared to negative control, which are in agreement with the Fred Docking scores reported in Table 2. Most importantly, the dendritic arborization of the primary neurons exposed to both nadolol and valproic acid was attenuated by the co-treatment with a selective TrkB inhibitor, ANA-12. ANA-12 blocks the neurotrophic actions of BDNF without compromising neuron survival. ANA-12 was shown in animal models to penetrate the BBB and block the cognitive effects associated with TrkB activation. TrkB agonists can activate the TrkB/BDNF pathways, which has been linked to cognitive effects based on the literature. However, in vitro primary neuron activation does not guarantee cognitive effects because the BBB penetration and brain bioavailability of componds are critical. However, studies have shown that 7,8-DHF-treated 5xFAD mice demonstrated cognitive improvement [50] and overexpression of BDNF [51]; we believe that compounds activating the TrkB/BDNF pathway with high brain bioavailability may have potential cognitive effects.

### 2.4. Detailed Analysis of TrkB and Top Candidate Interactions

TrkB signaling has been explored as a therapeutic target for neurological and psychiatric disorders [29,38,52]. However, the development of TrkB agonists has not made notable progress, especially with small molecule agonists. In addition, it is known that the binding location of TrkB agonists affects the regulation of the types of disorders [42]. Therefore, it is important to elucidate the interactions of TrkB binders and TrkB. Here, we will investigate the interaction details of the top candidates based on computed binding affinities: nadolol (ΔΔG = −8.13 kcal/mol), valproic acid (ΔΔG = −4.83 kcal/mol), and dicaffeoylquinic acid (ΔΔG = −7.63 kcal/mol). These binding energies were calculated using FRED docking scores based on the Chemgauss4 scoring function. The Chemgauss4 scoring function employs Gaussian smoothed potentials to evaluate the complementarity of ligand poses within the active site, focusing on interactions such as shape complementarity, hydrogen bonding (both with the protein and implicit solvent), and metal–chelator interactions. Consequently, our top candidate, nadolol, was selected based on its strong shape complementarity with the binding site. Additionally, nadolol forms the most hydrogen bonds with crucial active site residues, enhancing its binding stability. These components contribute to its higher docking scores. As a positive reference, we also provided the binding pocket analysis of 7,8-dihydroxyflavone (7,8-DHF), a small molecule that acts as a selective agonist to the TrkB receptor, specifically binding to its extracellular domain D5 [53,54,55]. Our computed binding affinity of 7,8-DHF is ΔΔG = −6.27 kcal/mol (Table 1 and Table 2), which is comparable to our top compounds, nadolol and dicaffeoylquinic acid.

**Figure 4:**
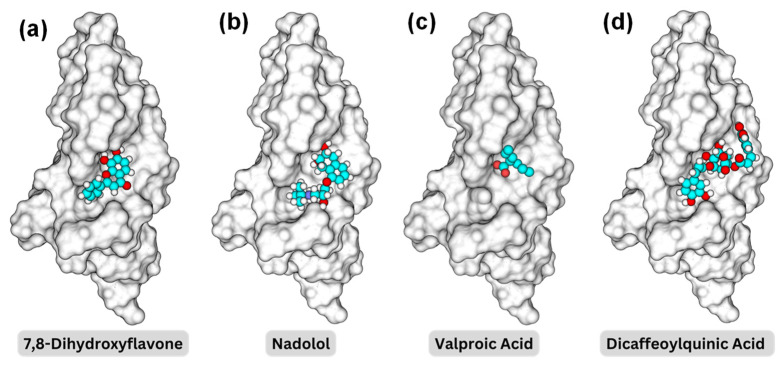
Binding pocket representations for (**a**) 7,8-dihydroxyflavone; (**b**) nadolol; (**c**) valproic acid; and (**d**) dicaffeoylquinic acid. The protein surface is shown in a transparent gray, while the molecules are displayed in space-filling models with carbon in cyan, oxygen in red, nitrogen in blue, and hydrogen in white.

Figure 4 illustrates the 3D structures of the TrkB-D5 domain in complex with four different ligands: 7,8-DHF, nadolol, valproic acid, and dicaffeoylquinic acid, highlighting the spatial arrangement and interactions between the protein and the ligand of the binding pocket BP1. In each subfigure, the TrkB-D5 structure is rendered as a grey surface representation to depict the overall shape and binding pocket. The small molecule is shown as a ball-and-stick model with carbon atoms in cyan, oxygen atoms in red, and hydrogen atoms in white. To reveal the binding mechanism between the potential agonist and TrkB-D5 receptor, we first analyzed the interactions between TrkB-D5 (chain X) and NT-4/5 (chain A) in the complex of PDBID: 1HCF.

**Figure 5:**
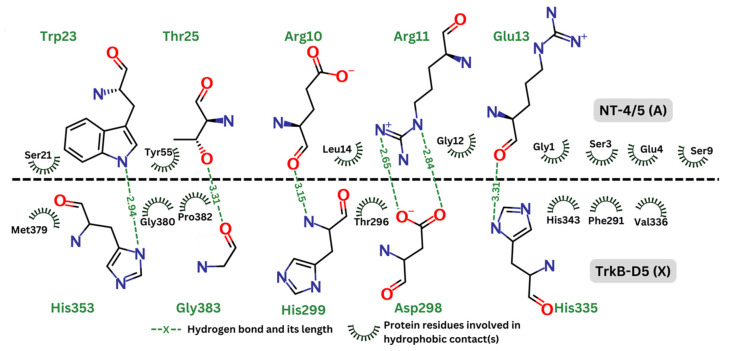
Contact map between TrkB-D5 (chain X) and NT-4/5 (chain A) in complex of PDBID: 1HCF.

**Figure 6:**
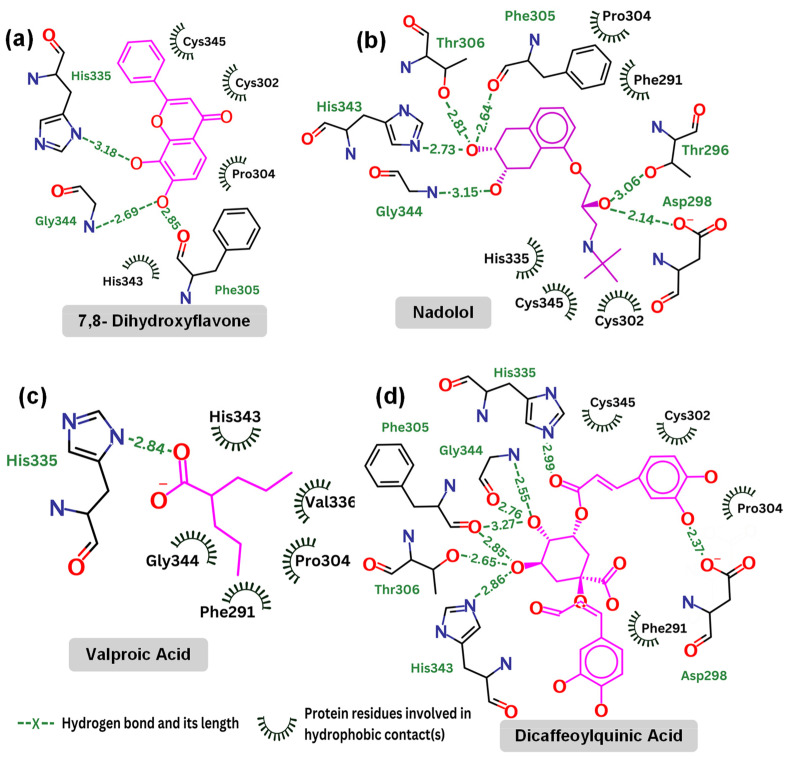
Contact map: (**a**) 7,8-dihydroxyflavone (kcal/mol); (**b**) nadolol (kcal/mol); (**c**) valproic acid (kcal/mol); and (**d**) dicaffeoylquinic Acid (kcal/mol).

Figure 5 depicts the hydrogen bonds as well as the residues involved in hydrophobic effect. This analysis was generated by Ligplot+ [56] and re-rendered using Marvin [57]. In the contact map, the hydrogen bonds are represented by green dashed lines, indicating the distance between donor and acceptor atoms. These bonds are expected to be crucial for the stability and specificity of the interaction between TrkB-D5 and NT-4/5. Key residues forming the hydrogen bonds in TrkB-D5 include His353, Gly383, Asp298, and His335. The hydrophobic contacts are depicted by curved lines around the involved residues. These interactions contribute to the binding affinity and stabilization of the complex. Significant hydrophobic contacts include residues such as Met379, Gly380, Pro382, Thr296, His343, Phe291, and Val336 in the TrkB-D5 domain, interacting with various residues in NT-4/5. It is reasonable to expect that agonists will involve similar residues of the TrkB-D5 receptor in forming hydrogen bonds and hydrophobic contacts. Similar to the interaction analysis in the 1HCF complex, we also revealed the details of hydrogen bonds and hydrophobic contacts of the selected ligands in Figure 6.

Table 3 highlights the specific interactions between those compounds and the TrkB-D5 domain, including details on the hydrogen bonds, distances, and angles for each interaction. It is reported that 7,8-DHF forms several hydrogen bonds with residues Gly344, Phe305, and His335. Note that His335 is also involved in the hydrogen bonds of TrkB-D5 and NT-4/5 complex. 7,8-DHF’s binding energy of −6.27 kcal/mol indicates moderate binding affinity. This relatively higher (less favorable) binding energy is attributed to the fewer and less optimal hydrogen bond interactions compared to other ligands. The most effective agonist among the compounds investigated is nadolol, with a binding energy calculated at −8.13 kcal/mol. This small molecule exhibits extensive hydrogen bonding with key residues Asp298, Thr296, Gly344, His343, Thr306, and Phe305. The presence of these five hydrogen bonds is responsible for the lower binding energy between nadolol and the TrkB-D5 receptor. Nadolol and 7,8-DHF share two common hydrogen bonds involving residues Gly344 and Phe305. Although the Phe305 hydrogen bond in nadolol (2.64 Å, 57.2°) appears to be weaker than the corresponding bond in 7,8-DHF (2.85 Å, 110.6°), the higher number of hydrogen bonds in nadolol contributes to its stronger binding affinity. Specifically, nadolol features strong hydrogen bonds with His343 (2.73 Å, 118.4°) and Asp298 (2.14 Å, 96.9°), which significantly enhance its binding stability and affinity.

Valproic acid forms a single hydrogen bond with His335 of TrkB-D5, resulting in a binding affinity of −4.83 kcal/mol, which is not very promising. Notably, both 7,8-DHF and dicaffeoylquinic acid also form hydrogen bonds with His335, but these bonds are weaker in terms of distance and angle (see Table 3). Additionally, His335 of TrkB-D5 forms a hydrogen bond with Glu13 of NT-4/5 in their complex (see Figure 4). This suggests that His335 plays an important role in the binding mechanism of TrkB-D5 with its ligands. However, it may not be crucial for strengthening the binding affinity, as evidenced by 7,8-DHF, which has a weak hydrogen bond with His335 yet still exhibits low binding energy.

Dicaffeoylquinic acid is the second strongest binder after nadolol, with an estimated binding energy of −7.63 kcal/mol, attributed to seven hydrogen bonds. Both dicaffeoylquinic acid and nadolol form hydrogen bonds with the same residues of TrkB-D5, including Asp298, Gly344, Phe305, and His343. While dicaffeoylquinic acid forms a weaker hydrogen bond with Asp298 compared to nadolol, its hydrogen bond with Gly344 is much stronger. However, the binding energy of dicaffeoylquinic acid (−7.63 kcal/mol) is less competitive than that of nadolol (−8.13 kcal/mol). Therefore, it can be concluded that forming a strong hydrogen bond with Asp298 is crucial for effective binding to TrkB. In support of this, Asp298 plays an important role in the hydrogen bond interactions between TrkB-D5 and NT-4/5 (Figure 4). All of the key amino acids responsible for the hydrogen bonding and hydrophobic interactions of the binding site regions (BP1 to BP5) within the TrkB-D5 protein are shown in Table S2. Each binding site region is defined by a unique set of amino acids that contribute to the overall stability and function of the protein–ligand complex.

To futher confirm the stability of these top compounds, we have carried out molecular simulations using the Desmond package (MD) from Schrodinger v2024-1. These simulations were performed to understand the stability and conformational changes of the protein–ligand complexes over a 10 ns period. The TIP3P model was used for the solvent, and the OPSL4 force field was applied for the simulations. Data were recorded at intervals of 10 ps. After 10 ns, the RMSD values for both the protein and ligands were mostly within the acceptable limit of 2.5 Å, indicating stable complexes throughout the simulation period, as shown in Figure S3.

## 3. Discussion

Our MSN screening nanoplatform was based on the specific ligand–receptor interactions, where the outcome of screening experiments is receptor binders, while the biological functions are not guaranteed. Therfore, bilogical activity tests are normally performed to confirm the identification of compounds. In addition, for compound screening, the specificity of the interaction with the target is critical. To ensure the specificity of TrkB binders, we applied two strategies: (1) a control cell without the particular receptors and (2) blocking the receptor with a known blocker; as controls. For example, for TrkB binder identification, cells overexpressing TrkB and cells without TrkB (control) were used. In addition, a well known inhibitor ANA-12 was also used to block the TrkB. For identified TrkB binders, dendritic arborization assay was used to evaluate the biological activities of TrkB binders from the screening experiments. Dendritic arborization is a functionally relevant in vitro endpoint as it reflects the potential to modulate synaptic plasticity, which serves as an indicator for TrkB activation [58].

To develop the docking methods for the evaluation of the binding location and binding affinity of the identified compounds, we selected TrkB-D5 as the receptor for docking. TrkB-D5 is considered as a favorable target for neurological and psychiatric disorder agonists, which bind to this TrkB domain to mimic the binding of Brain-Derived Neurotrophic Factor (BDNF) [38,39,40,41,59]. Subsequently, we used the experimentally tested compound identified by MSN from Gotu kola plant extracts to validate the docking methods. Our experimentally tested bioactive compound, 4-O-cafeoylquinic acid, showed high levels of binding affinity to all five binding sites, with much high binding in site 1. Although dicaffeopllauiric acid and castillicetin have slightly stronger binding to BP1 than 4-O-cafeoylquinic acid, the involvement of other binding sites is significantly lower than 4-O-cafeoylquinic acid. This trend is very similar to the Fred Docking scores of the known TrkB agonist, 7,8-dihydroxyflavone, where BP1 has high binding affinity, with the involvement of all other four binding sites. The synergy between the docking studies and the experimental observation suggests that our docking computation provides an effective tool to predict the activity of TrkB binders.

Subsequently, we applied a similar experimental screening process to the NIH clinical collection library and docking methods to the identified binders. The Fred Docking scores (in kcal/mol) of the identidied compounds from the MSN screening of the NIH library showed very similar binding behaviors to the TrkB binders from the Gotu kola plant extracts, where BP1 is the favorable binding site and only some compounds are involved in all of the binding sites. In addition, the binding affinities for the stronger binders were also very close such as dicaffeopllauinic acid (−7.63 kcal/mol) and 4-O-cafeoylquinic acid (−7.02 kcal/mol) for binders from plant extracts and nadolol (−8.13 kcal/mol) and clofazimine (−7.36 kcal/mol) for binders from the NIH library. These results suggest that the MSN screening process is highly reliable and applicable to different screening sources. In addition, the docking methods can be effectively used to predict the binding affinity and binding sites.

To further validate the docking methods, the biological activities of two candidates (nadolol—the strongest binding affinity, and valproic acid—medium binding affinity) were experimentally tested. The increased dendritic arborization of the primary neuron treated by nadool was similar to that of BDNF, but treatment with valprioc acid was less effective. These results suggested the correlation of the Fred Docking scores and the biological activities of the binders. In addition, the dendritic arborization of the primary neurons exposed to both nadolol and valproic acid was attenuated by the co-treatment with a selective TrkB inhibitor, ANA-12. This observation indicated the direct involvement of TrkB activation and further proved the syngery of the docking methods and the MSN screening for the identification of potenrial TrkB agonists.

Detailed binding studies also suggest that the strength and number of hydrogen bonds significantly influence the binding affinity of ligands to the TrkB-D5 domain. Specifically, the presence of strong hydrogen bonds with key residues such as Asp298 and His343 is crucial for achieving low binding energies and thus high binding affinity. Additionally, while His335 plays a role in the binding mechanism, it may not be critical for enhancing binding strength, as evidenced by the moderate affinity of 7,8-DHF and the weak binding of valproic acid. Overall, these findings highlight the importance of specific hydrogen bond interactions in determining the binding efficacy of potential TrkB-D5 agonists.

## 4. Materials and Methods

### 4.1. Materials

Chemicals and reagents were mainly purchased from VWR unless otherwise indicated: ferric chloride (VWR, 98% purity, St. Louis, MO, USA), sodium acetate (99%), ethylene glycol, sodium polyacrylate solution (Sigma Aldrich (St. Louis, MO, USA), 45% water, MW = 1200), RPMI 1640 medium (Invitrogen, Carlsbad, CA, USA), fetal bovine serum (FBS, Thermo Scientific, Waltham, MA, USA), penicillin/streptomycin (Thermo Scientific), Tris-HCl, NaCl (>99%), MgCl_2_ (≥98%), CaCl_2_ (≥99%), KCl (>99%), ammonium acetate (>99%), benzamidine hydrochloride (>99%), EDTA (≥98%), phenylmethanesulfonylfluoride (PMSF ≥ 98.5%), and geneticin (G418).

### 4.2. Magnetic Screening Nanoplatform (MSN)

MSN using TrkB as a screening target was prepared using our previously reported protocols [32]. Briefly, cell membrane fragments were first isolated from SH-SY5Y cells overexpressing TrkB and TrkB-null cells treated with hypotonic buffers. Subsequently, 3 mL of cell membrane fragment Bis-Tris buffer (20 mM, pH 7.2) solution from 107 cells was mixed with 1.0 mL of sterilized 1.0 mg/mL polyacrylic acid-coated iron oxide nanoclusters. The mixture was vortexed briefly and incubated on ice for 30 min at room temperature. Then, the mixture was tip sonicated for 120 s (following the method 27% amplitude, 5 s on, and 5 s off) using a Branson Digital Sonifier with a micro one-eighth-inch tip. After characterization, MSN with TrkB was stored at 4 °C until further experiments were conducted.

### 4.3. Screening of NIH Library

We have previously established the screening conditions for hot water plant extract and small molecule DMSO mixtures, and compound elution protocols using MSN with TrkB [32]. Here, we used the NIH clinical collection library as the screening source that contains 708 compounds used in human clinical trials with known therapeutic functions, biological mechanisms of action, and available structural and physicochemical properties. To perform the fishing experiment, a mixture of compounds from the library was prepared by combining 1 µL of each compound (10 mM). The mixture was then diluted with ammonium acetate buffer (50 mM, pH 7.4) 100 times followed addition of 250 µL of MSN. The mixture was incubated for 20 min at 37 °C to facilitate ligand binding. Then, the MSN- with bound compounds were magnetically separated from the mixture and washed three times using ammonium acetate buffer (250 µL, 50 mM, pH 7.4). Finally, MSN-bound compounds were eluted with 250 µL of methanol/ammonium acetate buffer (1/9, 5/5, and 9/1 *v*/*v*) to release compounds of different polarities. The elution profiles were analyzed using a Waters Xevo G2xs quadrupole-time-of-flight mass spectrometry with an iclass ultra-high-performance liquid chromatography system.

### 4.4. Docking Computation of TrkB and Its Agonists

In this study, we used Fred [37], a computational docking tool from OpenEye, to perform all docking calculations. We first employed the flipper utility from the OpenEye OMEGA module [60] to systematically identify and assign configurations to stereocenters with unspecified stereochemistry within our compounds. This process was initiated by providing the input file containing SMILES representations of compounds with indeterminate stereochemistry. The flipper tool was then used to generate isomers for these compounds, each of which was labeled with a distinct identifier to ensure their uniqueness. This process allows for the enumeration of five distinct isomers per stereocenter. Then, we determined the predominant tautomeric and protonation states for each isomer at physiological pH (7.4), a crucial step for accurately representing the chemical structures under biological conditions. We utilized the tautomeric application from the OpenEye QUACPAC module [61] for this calculation, where the input is the SMILES file containing all stereoisomeric forms from the previous step and the output is the most stable tautomeric and protonation state for each isomer.

To prepare the initial ligand pose for the docking simulations, we utilized the oeomega tool [60] from the OpenEye software suite 2024.1.0. This process transforms two-dimensional SMILES representations of the ligands into three-dimensional structures ready for docking. The output is saved in SDF format. For this ligand pose generation, the creation of conformers is permitted without strict stereochemical constraints, which is relevant when the stereochemistry is undefined or variable. To ensure the accuracy of our docking simulations, it is crucial to begin with a fully prepared receptor structure. This preparation entails adding any missing protons or residues that may be absent from the initial protein data file. Completing the receptor’s structure provides a more reliable foundation for subsequent simulations and analyses. For this purpose, the profix tool from JACKAL software [62] is employed. Specifically, we selected the “-fix 0” option, designed to address missing atoms, including protons, within the receptor without attempting to reconstruct missing residues. This focused approach ensures that our receptor models are proton-complete, a crucial factor for accurate hydrogen bond network predictions.

To determine the potential binding sites of TrkB, we leverage the capabilities of the OEDocking Graphical User Interface (GUI) [37] developed by OpenEye. The OEDocking, renowned for its intuitive design and advanced computational algorithms, allows us to efficiently identify sites on the protein surface that are likely to accommodate ligand molecules. To begin, we loaded the three-dimensional structure of our target protein into the OEDocking GUI. The software then performs a comprehensive analysis of the protein’s surface, employing a combination of geometric and chemical heuristics to detect cavities that could serve as feasible binding sites based on the molecular cavity detection algorithms. Once the potential sites are identified, the OEDocking GUI provides an option to save these sites for further analysis. We utilized this feature to preserve the identified sites in a designated file format, which facilitates subsequent steps in our workflow, such as docking simulations. Lastly, we employed Fred to dock the chosen binding sites with the ligands, using the initial poses generated in preceding steps. The most favorable binding site regions are then ranked according to the Fred Docking scores.

### 4.5. Structures of Docked Compounds

In the context of docking simulations, our investigation targets the binding interactions of eight specific compounds derived from the Gotu Kola plant with the TrkB receptor [32]. Additionally, we are examining the binding mechanisms of the top 20 compounds as identified through experimental methods from the NIH repository. The SMILES representations of these compounds are listed in Table S1.

### 4.6. Biological Activity Evaluation of Top Compounds

The binding interactions of the TrkB receptor with the eight specific compounds derived from the Gotu Kola plant from our previous studies [32] showed the strong agreement of the binding score and experimental activities. Therefore, using the binding score as guidelines, we selected two additional compounds with distinct binding affinities from the NIH clinical collection library as top candidates for experimental verification, namely, nadolol and valproic acid. We performed the initial evaluation of these two compounds to evaluate their effects on dendritic arborization elongation using primary hippocampal neurons isolated from embryonic C57BL6 mice following the previously described method [62,63]. Briefly, hippocampal neurons were isolated from embryos on gestational day 18 and plated at a density of 130,000 cells in a 60 mm dish containing three glass coverslips. After 3 h, coverslips were flipped into 60 mm dishes containing mouse neural stem cell-derived glial cells. Co-culture continued for 14 days, at which point cells were treated with BDNF at 25 ng/mL, nadolol at 10 nM, and valproic acid at 500 µM. One week later, cells were fixed in 4% paraformaldehyde and stained with Anti-MAP2B. Immunostained neurons were imaged with a Zeiss ApoTome2 microscope, and blinded Sholl analyses were performed to assess dendritic complexity using the Fiji platform. The Sholl analysis was measured as intersections of neurites through concentric circles around the cell body moving out at 10 µm intervals. Here, BDNF treatment was used as a positive control. Additionally, the experiments were performed by the co-treatment with a selective TrkB inhibitor ANA-12. ANA-12 blocks the neurotrophic actions of BDNF without compromising neuron survival. ANA-12 co-treatment allowed one to confirm the involvement of TrkB activation in the neurite development.

## 5. Conclusions

In conclusion, we reported a combined computational and experimental approach to study the TrkB binders for potential neurological disorder treatment. The Fred Docking scores (in kcal/mol) of experimentally identified TrkB binders from docking simulation displayed strong agreement with the top candidates. The experimentally tested top candidates, such as 4-O-QCA and nadolol, effectively increased the dendritic arborization of primary hippocampal neurons isolated from AD-mouse models. From the docking simulation, these top candidates showed the high affinity of the most favorable binding site (BP1), with the involvement of the other four binding sites. This observation is also true for the best-characterized, thoroughly in vivo-tested, small molecule TrkB agonist, 7,8-dihydroxyflavone. These studies not only confirmed the effectiveness of our magnetic drug screening platform in identifying biological active compounds from mixtures but also showed the feasibility of using docking simulation to predict the biological activities of compounds. Particularly, our detailed interaction analysis demonstrated that specific hydrogen bonds, especially those involving key residues such as Asp298 and His343, are crucial for strong binding affinities. This combined experimental and computation approach is poised to explore the BDNF/TrkB pathway as a viable target for drug development. This study will lead to a list of TrkB binders with potential biological activities. Our future studies will focus on in vivo efficacy studies in animal models. Depending on the structures of the compounds, further structural alteration or formulation lead compounds may be needed for BBB penetration and enhanced brain bioavailability in order to be advanced to the next phase of drug development. Furthermore, once compounds stimulating the BDNF/TrkB pathway are validated, different mouse models of other neurodegenerative diseases can further evaluate these compounds. Finally, the developed drug-screening assay will be transferable to other transmembrane targets related to neurodegenerative diseases. Our combined approach will not only greatly benefit drug discovery processes using TrkB transmembrane proteins as targets but also allow for the evaluation and validation of any previously reported TrkB binders.

## Figures and Tables

**Table 1:** Fred Docking scores (in kcal/mol) for all five different binding sites for compounds from Gotu Kola extracts. BP stands for binding site. The notation “-” denotes that the software failed to generate the pose because the docking score is out of the acceptable range. Here, the known TrkB agonist, 7,8-dihydroxyflavone, was included for comparison.

Compound Names	BP 1	BP 2	BP3	BP4	BP5
Dicaffeopllauinic Acid	−7.63	0.74	−3.06	1.15	0.2
Castillicetin	−7.32	2.59	−3.71	0.02	−2.10
O-Caffeoylquinic Acid	−7.02	−3.77	−5.82	−4.14	−4.45
Stigmasterol	−6.70	-	0.85	-	3.63
7,8-dihydroxyflavone	−6.27	−2.26	−5.12	−3.91	−3.26
Quercetin	−5.86	−3.35	−5.09	−3.25	−3.20
Pomolic Acid	−4.99	5.91	−0.27	4.66	1.60
Madecassic Acid	−4.89	5.94	−1.30	8.57	1.90
Naringin	−3.52	-	-	-	-

**Table 2:** Fred Docking scores (in kcal/mol) for all five different binding sites for compounds from the NIH library. BP stands for binding site. The notation “-” denotes that the software failed to generate the pose because the docking score is out of the acceptable range. Here, the known TrkB agonist, 7,8-dihydroxyflavon, was included as a comparison.

Compound Names	BP1	BP2	BP3	BP4	BP5
Nadolol	−8.13	−3.0	−4.75	−2.96	−4.95
Benproperine Phosphate	−7.63	−0.51	−4.49	−2.44	−3.7
Clofazimine	−7.36	6.46	2.77	-	−4.79
Brimonidine	−7.32	−4.77	−4.56	−4.51	−5.15
Amiodarone Hydrochloride	−6.95	-	-	-	-
3,5,3′-Triiodothyronine	−6.67	−0.95	−4.69	−3.00	−3.13
7,8-dihydroxyflavone	−6.27	−2.26	−5.12	−3.91	−3.26
Mestranol	−6.18	−0.91	−3.45	−1.81	−1.48
Levonorgestrel	−5.86	−0.08	−3.76	−2.57	−1.22
Ondansetron	−5.84	−0.73	−3.53	−3.68	−3.29
Vecuronium Bromide	−5.16	-	3.64	-	9.9
Raloxifene HCl	−5.54	9.62	2.24	7.45	5.01
Oxaprozin	−5.00	−1.2	−3.53	−3.07	−3.89
Valproic Acid	−4.83	−3.06	−3.80	−2.96	−3.28
Deferiprone	−3.66	−3.79	-	-	-
Diazepam	−4.4	−1.31	−3.13	−1.39	−2.57
Loperamide Hydrochloride	−4.50	8.24	−0.32	7.97	3.98

**Table 3:** Hydrogen analysis between the top candidates and TrkB-5 domain. The first atom in the hydrogen bond representation is from the considered ligand. The second component, specified with the residue ID it is located in, is the acceptor (or donor) from TrkB-D5.

Hydrogen Bond	Distance (Å)	Angle (°)
7,8-dihydroxyflavone		
O3-H…N(Gly344)	2.69	46.0
O3-H…O(Phe305)	2.85	110.6
O4-H…ND1(His335)	3.18	45.6
Nadolol		
O21-H…OD2(Asp298)	2.14	96.9
O21…H-OG1(Thr296)	3.06	62.5
O20-H…N(Gly344)	3.15	82.2
O19…H-ND1(His343)	2.73	118.4
O19…H-OG1(Thr306)	2.8	61.3
O19-H…O(Phe305)	2.64	57.2
Valproic Acid		
O10…H-ND1(His335)	2.84	149.3
Dicaffeoylquinic Acid		
O32-H…OD2(Asp298)	2.37	45.4
O27…H-ND1(His335)	2.99	129.7
O35-H…O(Gly344)	2.76	134.7
O35…H-N(Gly344)	2.55	96.4
O35-H…O(Phe305)	3.27	65.2
O34-H…OG1(Thr306)	2.65	91.5
O34…H-ND1(His343)	2.86	122.2

## Data Availability

The original contributions presented in the study are included in the article/Supplementary Materials; further inquiries can be directed to the corresponding author/s.

## Supplementary Materials

The following supporting information can be downloaded at https://www.mdpi.com/article/10.3390/molecules29173992/s1, Figure S1: Compound identification process; Figure S2: Mass spectra of identified peaks for compound conformation; Figure S3: MD simulations of docked complexes; Table S1: Summary of 25 compound structures; and Table S2: TrkB-D5 binding site analysis.

## References

[1] Adkins-Jackson P.B., Belsky D.W. (2022). Alzheimer’s disease risk biomarkers: Progress and challenges. Lancet Healthy Longev..

[2] Yiannopoulou K.G., Anastasiou A.I., Zachariou V., Pelidou S.H. (2019). Reasons for Failed Trials of Disease-Modifying Treatments for Alzheimer Disease and Their Contribution in Recent Research. Biomedicines.

[3] Cummings J., Zhou Y., Lee G., Zhong K., Fonseca J., Cheng F. (2024). Alzheimer’s disease drug development pipeline: 2024. Alzheimer's Dement. Transl. Res. Clin. Interv..

[4] Mullane K., Williams M. (2020). Alzheimer’s disease beyond amyloid: Can the repetitive failures of amyloid-targeted therapeutics inform future approaches to dementia drug discovery?. Biochem. Pharmacol..

[5] Ferrer I., Marin C., Rey M.J., Ribalta T., Goutan E., Blanco R., Tolosa E., Marti E. (1999). BDNF and full-length and truncated TrkB expression in Alzheimer disease. Implications in therapeutic strategies. J. Neuropathol. Exp. Neurol..

[6] Numakawa T., Odaka H., Adachi N. (2018). Actions of Brain-Derived Neurotrophin Factor in the Neurogenesis and Neuronal Function, and Its Involvement in the Pathophysiology of Brain Diseases. Int. J. Mol. Sci..

[7] Lima Giacobbo B., Doorduin J., Klein H.C., Dierckx R., Bromberg E., de Vries E.F.J. (2019). Brain-Derived Neurotrophic Factor in Brain Disorders: Focus on Neuroinflammation. Mol. Neurobiol..

[8] Wang Z.H., Xiang J., Liu X., Yu S.P., Manfredsson F.P., Sandoval I.M., Wu S., Wang J.Z., Ye K. (2019). Deficiency in BDNF/TrkB Neurotrophic Activity Stimulates delta-Secretase by Upregulating C/EBPbeta in Alzheimer’s Disease. Cell Rep..

[9] Devi L., Ohno M. (2015). TrkB reduction exacerbates Alzheimer’s disease-like signaling aberrations and memory deficits without affecting beta-amyloidosis in 5XFAD mice. Transl. Psychiatry.

[10] Jiao S.S., Shen L.L., Zhu C., Bu X.L., Liu Y.H., Liu C.H., Yao X.Q., Zhang L.L., Zhou H.D., Walker D.G. (2016). Brain-derived neurotrophic factor protects against tau-related neurodegeneration of Alzheimer’s disease. Transl. Psychiatry.

[11] Ng T.K.S., Ho C.S.H., Tam W.W.S., Kua E.H., Ho R.C. (2019). Decreased Serum Brain-Derived Neurotrophic Factor (BDNF) Levels in Patients with Alzheimer’s Disease (AD): A Systematic Review and Meta-Analysis. Int. J. Mol. Sci..

[12] Amidfar M., de Oliveira J., Kucharska E., Budni J., Kim Y.K. (2020). The role of CREB and BDNF in neurobiology and treatment of Alzheimer’s disease. Life Sci..

[13] Atasoy I.L., Dursun E., Gezen-Ak D., Metin-Armagan D., Ozturk M., Yilmazer S. (2017). Both secreted and the cellular levels of BDNF attenuated due to tau hyperphosphorylation in primary cultures of cortical neurons. J. Chem. Neuroanat..

[14] Rosa E., Mahendram S., Ke Y.D., Ittner L.M., Ginsberg S.D., Fahnestock M. (2016). Tau downregulates BDNF expression in animal and cellular models of Alzheimer’s disease. Neurobiol. Aging.

[15] Xiang J., Wang Z.H., Ahn E.H., Liu X., Yu S.P., Manfredsson F.P., Sandoval I.M., Ju G., Wu S., Ye K. (2019). Delta-secretase-cleaved Tau antagonizes TrkB neurotrophic signalings, mediating Alzheimer’s disease pathologies. Proc. Natl. Acad. Sci. USA.

[16] Giuffrida M.L., Copani A., Rizzarelli E. (2018). A promising connection between BDNF and Alzheimer’s disease. Aging.

[17] Ando S., Kobayashi S., Waki H., Kon K., Fukui F., Tadenuma T., Iwamoto M., Takeda Y., Izumiyama N., Watanabe K. (2002). Animal model of dementia induced by entorhinal synaptic damage and partial restoration of cognitive deficits by BDNF and carnitine. J. Neurosci. Res..

[18] Fischer D.L., Sortwell C.E. (2019). BDNF provides many routes toward STN DBS-mediated disease modification. Mov. Disord..

[19] Zhang F., Kang Z., Li W., Xiao Z., Zhou X. (2012). Roles of brain-derived neurotrophic factor/tropomyosin-related kinase B (BDNF/TrkB) signalling in Alzheimer’s disease. J. Clin. Neurosci..

[20] Pilakka-Kanthikeel S., Atluri V.S., Sagar V., Saxena S.K., Nair M. (2013). Targeted brain derived neurotropic factors (BDNF) delivery across the blood-brain barrier for neuro-protection using magnetic nano carriers: An in-vitro study. PLoS ONE.

[21] Jang S.W., Liu X., Yepes M., Shepherd K.R., Miller G.W., Liu Y., Wilson W.D., Xiao G., Blanchi B., Sun Y.E. (2010). A selective TrkB agonist with potent neurotrophic activities by 7,8-dihydroxyflavone. Proc. Natl. Acad. Sci. USA.

[22] Todd D., Gowers I., Dowler S.J., Wall M.D., McAllister G., Fischer D.F., Dijkstra S., Fratantoni S.A., van de Bospoort R., Veenman-Koepke J. (2014). A Monoclonal Antibody TrkB Receptor Agonist as a Potential Therapeutic for Huntington’s Disease. PLoS ONE.

[23] Liu X., Chan C.B., Qi Q., Xiao G., Luo H.R., He X.L., Ye K.Q. (2012). Optimization of a Small Tropomyosin-Related Kinase B (TrkB) Agonist 7,8-Dihydroxyflavone Active in Mouse Models of Depression. J. Med. Chem..

[24] Liu X., Obianyo O., Chan C.B., Huang J., Xue S., Yang J.J., Zeng F., Goodman M., Ye K. (2014). Biochemical and biophysical investigation of the brain-derived neurotrophic factor mimetic 7,8-dihydroxyflavone in the binding and activation of the TrkB receptor. J. Biol. Chem..

[25] Chen L., Gao X., Zhao S., Hu W., Chen J. (2015). The Small-Molecule TrkB Agonist 7, 8-Dihydroxyflavone Decreases Hippocampal Newborn Neuron Death After Traumatic Brain Injury. J. Neuropathol. Exp. Neurol..

[26] Zhang Z., Liu X., Schroeder J.P., Chan C.B., Song M., Yu S.P., Weinshenker D., Ye K. (2014). 7,8-dihydroxyflavone prevents synaptic loss and memory deficits in a mouse model of Alzheime’s disease. Neuropsychopharmacology.

[27] Massa S.M., Yang T., Xie Y.M., Shi J., Bilgen M., Joyce J.N., Nehama D., Rajadas J., Longo F.M. (2010). Small molecule BDNF mimetics activate TrkB signaling and prevent neuronal degeneration in rodents. J. Clin. Investig..

[28] Edelbrock A.N., Alvarez Z., Simkin D., Fyrner T., Chin S.M., Sato K., Kiskinis E., Stupp S.I. (2018). Supramolecular Nanostructure Activates TrkB Receptor Signaling of Neuronal Cells by Mimicking Brain-Derived Neurotrophic Factor. Nano Lett..

[29] Casarotto P.C., Girych M., Fred S.M., Kovaleva V., Moliner R., Enkavi G., Biojone C., Cannarozzo C., Sahu M.P., Kaurinkoski K. (2021). Antidepressant drugs act by directly binding to TRKB neurotrophin receptors. Cell.

[30] Jetsonen E., Didio G., Winkel F., Llach Pou M., Boj C., Kuczynski-Noyau L., Võikar V., Guirado R., Taira T., Lauri S.E. (2023). Activation of TrkB in Parvalbumin interneurons is required for the promotion of reversal learning in spatial and fear memory by antidepressants. Neuropsychopharmacology.

[31] Sherwood J., Sowell J., Beyer N., Irvin J., Stephen C., Antone A.J., Bao Y.P., Ciesla L.M. (2019). Cell-membrane coated iron oxide nanoparticles for isolation and specific identification of drug leads from complex matrices. Nanoscale.

[32] Arituluk Z.C., Horne J., Adhikari B., Steltzner J., Mansur S., Ahirwar P., Velu S.E., Gray N.E., Ciesla L.M., Bao Y. (2021). Identification of TrkB binders from complex matrices using a magnetic drug screening nanoplatform. ACS Appl. Bio Mater..

[33] Obergrussberger A., Friis S., Bruggemann A., Fertig N. (2021). Automated patch clamp in drug discovery: Major breakthroughs and innovation in the last decade. Expert Opin. Drug Discov..

[34] Ciesla L., Moaddel R. (2016). Comparison of analytical techniques for the identification of bioactive compounds from natural products. Nat. Prod. Rep..

[35] Ciesla L., Okine M., Rosenberg A., Dossou K.S.S., Toll L., Wainer I.W., Moaddel R. (2016). Development and characterization of the alpha3beta4alpha5 nicotinic receptor cellular membrane affinity chromatography column and its application for on line screening of plant extracts. J. Chromatogr. A.

[36] Thornburg C.C., Britt J.R., Evans J.R., Akee R.K., Whitt J.A., Trinh S.K., Harris M.J., Thompson J.R., Ewing T.L., Shipley S.M. (2018). NCI Program for Natural Product Discovery: A Publicly-Accessible Library of Natural Product Fractions for High-Throughput Screening. ACS Chem. Biol..

[37] McGann M. (2011). FRED pose prediction and virtual screening accuracy. J. Chem. Inf. Model..

[38] Zagrebelsky M., Korte M. (2024). Are TrkB receptor agonists the right tool to fulfill the promises for a therapeutic value of the brain-derived neurotrophic factor?. Neural Regen. Res..

[39] Ademuwagun I.A., Oduselu G.O., Rotimi S.O., Adebiyi E. (2023). Pharmacophore-Aided Virtual Screening and Molecular Dynamics Simulation Identifies TrkB Agonists for Treatment of CDKL5-Deficiency Disorders. Bioinform. Biol. Insights.

[40] Enkavi G., Girych M., Moliner R., Vattulainen I., Castrén E. (2024). TrkB transmembrane domain: Bridging structural understanding with therapeutic strategy. Trends Biochem. Sci..

[41] Pattarawarapan M., Burgess K. (2003). Molecular Basis of Neurotrophin−Receptor Interactions. J. Med. Chem..

[42] Ibáñez C.F. (1998). Emerging themes in structural biology of neurotrophic factors. Trends Neurosci..

[43] Banfield M.J., Naylor R.L., Robertson A.G., Allen S.J., Dawbarn D., Brady R.L. (2001). Specificity in Trk receptor: Neurotrophin interactions: The crystal structure of TrkB-d5 in complex with neurotrophin-4/5. Structure.

[44] Berman H., Henrick K., Nakamura H. (2003). Announcing the worldwide protein data bank. Nat. Struct. Mol. Biol..

[45] Beaman E.E., Bonde A.N., Larsen S.M.U., Ozenne B., Lohela T.J., Nedergaard M., Gíslason G.H., Knudsen G.M., Holst S.C. (2023). Blood–brain barrier permeable β-blockers linked to lower risk of Alzheimer’s disease in hypertension. Brain.

[46] CuraSen Therapeutics I.s.P. (2023). A Study of CST-2032 and CST-107 in Subjects with Mild Cognitive Impairment or Mild Dementia Due to Parkinson's or Alzheimer's Disease.

[47] Dedoni S., Marras L., Olianas M.C., Ingianni A., Onali P. (2019). Downregulation of TrkB Expression and Signaling by Valproic Acid and Other Histone Deacetylase Inhibitors. J. Pharmacol. Exp. Ther..

[48] Mikitsh J.L., Chacko A.M. (2014). Pathways for small molecule delivery to the central nervous system across the blood-brain barrier. Perspect. Med. Chem..

[49] Laurens C., Abot A., Delarue A., Knauf C. (2019). Central Effects of Beta-Blockers May Be Due to Nitric Oxide and Hydrogen Peroxide Release Independently of Their Ability to Cross the Blood-Brain Barrier. Front. Neurosci..

[50] Chen C., Wang Z., Zhang Z., Liu X., Kang S.S., Zhang Y., Ye K. (2018). The prodrug of 7,8-dihydroxyflavone development and therapeutic efficacy for treating Alzheimer’s disease. Proc. Natl. Acad. Sci. USA.

[51] de Pins B., Cifuentes-Díaz C., Farah A.T., López-Molina L., Montalban E., Sancho-Balsells A., López A., Ginés S., Delgado-García J.M., Alberch J. (2019). Conditional BDNF Delivery from Astrocytes Rescues Memory Deficits, Spine Density, and Synaptic Properties in the 5xFAD Mouse Model of Alzheimer Disease. J. Neurosci..

[52] Moliner R., Girych M., Brunello C.A., Kovaleva V., Biojone C., Enkavi G., Antenucci L., Kot E.F., Goncharuk S.A., Kaurinkoski K. (2023). Psychedelics promote plasticity by directly binding to BDNF receptor TrkB. Nat. Neurosci..

[53] Chitranshi N., Gupta V., Kumar S., Graham S.L. (2015). Exploring the molecular interactions of 7, 8-dihydroxyflavone and its derivatives with TrkB and VEGFR2 proteins. Int. J. Mol. Sci..

[54] Chiang N.-N., Lin T.-H., Teng Y.-S., Sun Y.-C., Chang K.-H., Lin C.-Y., Hsieh-Li H.M., Su M.-T., Chen C.-M., Lee-Chen G.-J. (2021). Flavones 7, 8-DHF, quercetin, and apigenin against Tau toxicity via activation of TRKB signaling in ΔK280 TauRD-DsRed SH-SY5Y cells. Front. Aging Neurosci..

[55] Chiu Y.-J., Lin T.-H., Chang K.-H., Lin W., Hsieh-Li H.M., Su M.-T., Chen C.-M., Sun Y.-C., Lee-Chen G.-J. (2022). Novel TRKB agonists activate TRKB and downstream ERK and AKT signaling to protect Aβ-GFP SH-SY5Y cells against Aβ toxicity. Aging.

[56] Laskowski R.A., Swindells M.B. (2011). LigPlot+: Multiple Ligand–Protein Interaction Diagrams for Drug Discovery.

[57] ChemAxon (2024). Marvin Was Used for Drawing, Displaying and Characterizing Chemical Structures, Substructures and Reactions.

[58] Cazorla M., Prémont J., Mann A., Girard N., Kellendonk C., Rognan D. (2011). Identification of a low-molecular weight TrkB antagonist with anxiolytic and antidepressant activity in mice. J. Clin. Investig..

[59] Hawkins P.C., Skillman A.G., Warren G.L., Ellingson B.A., Stahl M.T. (2010). Conformer generation with OMEGA: Algorithm and validation using high quality structures from the Protein Databank and Cambridge Structural Database. J. Chem. Inf. Model..

[60] OpenEye (2024). QUACPAC, Version 2.2.3.3.

[61] Xiang J.Z., Honig B. (2002). Jackal: A Protein Structure Modeling Package.

[62] Kaech S., Banker G. (2006). Culturing hippocampal neurons. Nat. Protoc..

[63] Long H.S., Stander M.A., Van Wyk B.E. (2012). Notes on the occurrence and significance of triterpenoids (asiaticoside and related compounds) and caffeoylquinic acids in Centella species. S. Afr. J. Bot..

