# Supplementary material for: A Combined Computational and Experimental Approach to Studying Tropomyosin Kinase Receptor B Binders for Potential Treatment of Neurodegenerative Diseases
Source: Molecules. 2024 Aug 23;29(17):3992. doi: 10.3390/molecules29173992 (PMC11396239; doi:10.3390/molecules29173992)
Supplement: Supplementary file 1 [file molecules-29-03992-s001.zip › molecules-3110145-supplementary.pdf]

Supporting Information

A Combined Computational and Experimental Approach to study TrkB binders for Potential Neurodegenerative Disease

Duc D. Nguyen,[1]* Shomit Mansur,[2] Lukasz Ciesla,[3] Nora E. Gray,[4] Shan Zhao,[5] Yuping Bao[2]*

1. Magnetic screening nanoplatform with TrkB as screening targets

To ensure the specificity of the identified TrkB binders, Control MSN prepared with parental TrkB-null cells were used as negative control for the fishing experiments and the binders were identified based on the elution time in ultrahigh-performance liquid chromatography (UPLC). The elution profiles of binders bound to MSN with TrkB and MSN without TrkB were compared. Peaks only present in the elution profiles from MSN with TrkB, but not in the elution profiles from MSN without TrkB were identified as TrkB specific binders.

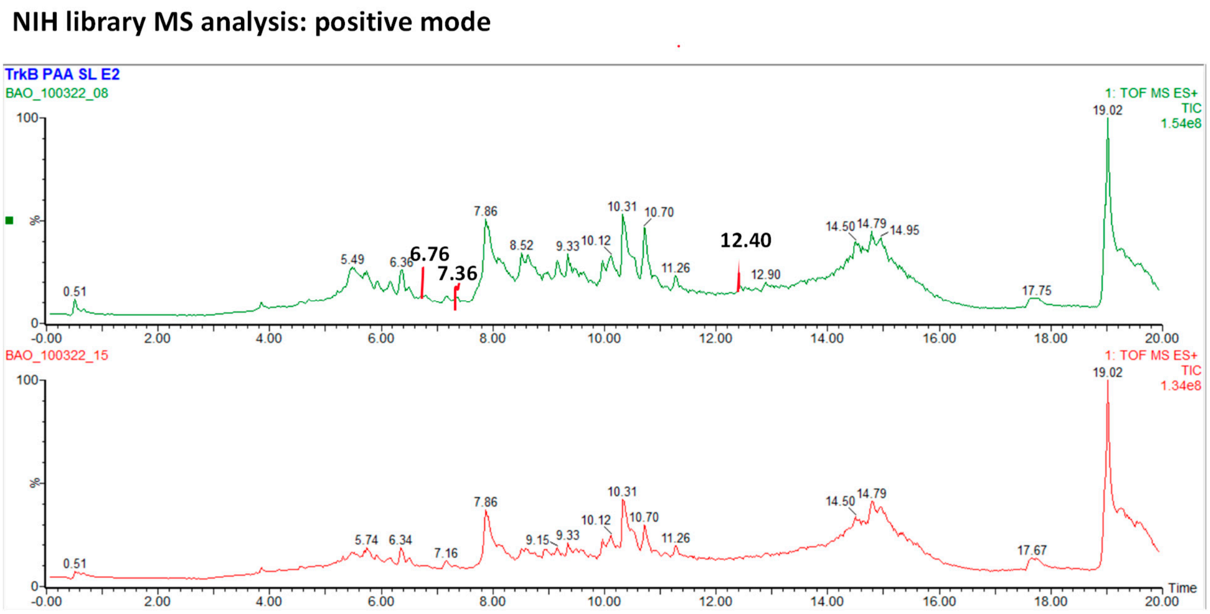

Figure S1. The elution profiles of compounds from MSN with TrkB (Top) and MSN without TrkB (bottom), where the differences were clarified as TrkB specific binders.

2. Compound identification of the binding compounds

To confirm identified compounds, the corresponding compound masses of the specific TrkB elution peaks were obtained in positive ionization mode using mass spectrometry (QTof-MS), as shown in Figure S2. The analysis of UPLC chromatograms of three elution peaks (6.76 min, 7.36 min and 12.40 min) led to a list of compounds. Then the detected compound masses were matched with the compound data from the NIH clinical collection library and possible matching compounds were listed (Figure S2).

MS at elution 7.36 min

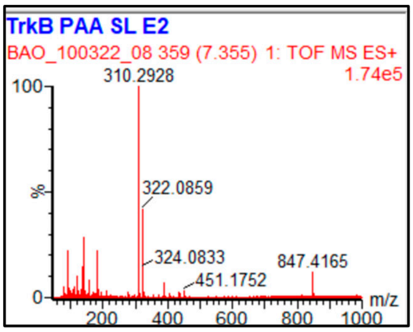

- At m/s peak 310.2928, possible matches
  - **Mestranol,** M_w= 310.4299 (Closest match)
  - **Levonorgestrel,** M_w= 312.2089 (-2H)
  - **Benproperine phosphate,** M_w= 309.4452 (+1H)
  - **Nadolol,** M_w= 309.4006 (+1H)
- At m/s peak 847.4165, possible matches
  - **Rifabutin,** M_w=847.4415

MS at elution time 6.76 min

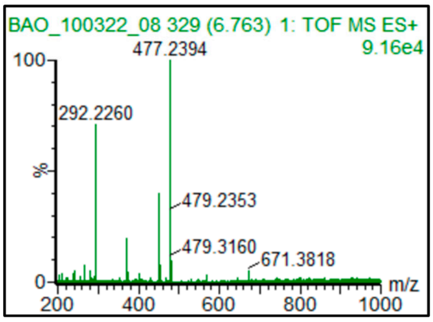

- At m/s peak 477.2394, possible matches
  - **Loperamide hydrochloride,** M_w= 477.2231
- At m/s peak 292.2260, possible matches
  - **Brimonidine,** M_w= 292.135 (closest match)
  - **Ondansetron,** M_w = 293.363 (-1H)
  - **Oxaprozin,** M_w=293.3166 (-1H)

MS at elution time 12.40 min

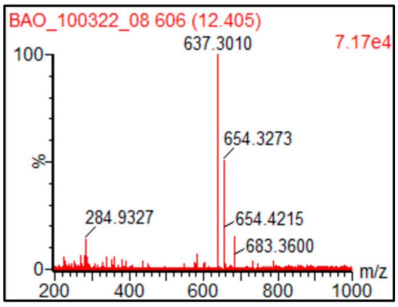

MS at elution time 12.90 min

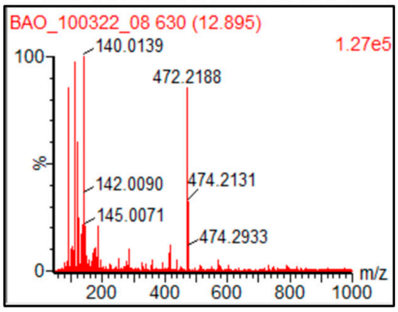

- At m/s peak 284.9327, possible matches
  - **Diazepam,** M_w= 284.74 (closest match)
- At m/s peak 637.3010,
  - **Vecuronium bromide,** M_w= 637.7308 (structural M_w)
- At m/s peak, 654.4215, possible matches
  - **Vecuronium bromide,** M_w= 637.7308 ($+H_2O-H^+$)
  - **3,5,3' – Triiodothyronine, M_w**= 650.9735 ($+3H^+$)
- At m/s peak 683.3600, possible matches
  - **Amiodarone hydrochloride,** M_w= 681.7725 (structural M_w)
- At m/s peak 140.0139, possible matches
  - **Deferiprone,** M_w= 139.1519 ($-1H^+$)
- At m/s peak 140.0139, possible matches
  - **Valproic acid,** M_w= 144.2114($+H^+$)
- At m/s peak 472.2188,
  - **Clofazimine,** M_w= 472.1222 (closest match)
  - **Raloxifene HCl,** M_w= 473.1661 ($+1H^+$)

Figure S2. Mass spectra of TrkB specific binders and possible matching compounds in the NIH clinical collection library.

3. Structures of docked compounds

In the context of docking simulations, our investigation targets the binding interactions of eight specific compounds derived from the Gotu Kola plant with the TrkB receptor [32]. Additionally, we are examining the binding mechanisms of the top 17 compounds as identified through experimental methods from the NIH repository. The identification and chemical structures of these compounds are meticulously detailed in Table .

Table S1: Summary of 25 compound structures: 8 from the Gotu Kola plant and 17 from the NIH repository.

Compound Names	SMILES	Source
Dicaffeoylquinic Acid	C1C(C(C(CC1(C(=O)O)OC(=O)C=CC2=CC(=C(C=C2)O)O)O)OC(=O)C=CC3=CC(=C(C=C3)O)O)O)O	Gotu Kola plant [32]
O-Caffeoylquinic Acid	C1C(C(C(CC1(C(=O)O)OC(=O)C=CC2=CC(=C(C=C2)O)O)O)O)O)O	Gotu Kola plant [32]
Madecassic acid	CC1CCC2(CCC3(C(=CCC4C3(CC(C5C4(CC(C(C5(C)CO)O)O)O)C)O)C)C2C1C)C)C(=O)O	Gotu Kola plant [32]
Castillicetin	C1=CC(=C(C=C1C=CC(=O)OC2=C(OC3=CC(=CC(=C3C2=O)O)O)C4=CC(=C(C=C4)O)O)O)O	Gotu Kola plant [32]
Quercetin	O=c1c(O)c(-c2ccc(O)c(O)c2)oc2cc(O)cc(O)c12	Gotu Kola plant [32]
Stigmasterol	CC[C@H](/C=C/[C@@H](C)[C@H]1CC[C@H]2[C@@H]3CC=C4C[C@@H](O)CC[C@]4(C)[C@H]3CC[C@@]21C)C(C)C	Gotu Kola plant [32]
Pomolic acid	C[C@@H]1CC[C@]2(C(=O)O)CC[C@]3(C)C(=CC[C@@H]4[C@@]5(C)CC[C@H](O)C(C)(C)[C@@H]5CC[C@]43C)[C@@H]2[C@]1(C)O	Gotu Kola plant [32]
Naringin	C[C@@H]1O[C@@H](O[C@H]2[C@H](Oc3cc(O)c4c(c3)O[C@H](c3ccc(O)cc3)CC4=O)O[C@H](CO)[C@@H](O)[C@@H]2O)[C@H](O)[C@H](O)[C@H]1O	Gotu Kola plant [32]
Mestranol	COc1ccc2[C@H]3CC[C@@]4(C)[C@@H](CC[C@@]4(O)C#C)[C@@H]3CCc2c1	NIH repository
Levonorgestrel	CC[C@]12CC[C@H]3[C@@H](CCC4=CC(=O)CC[C@H]34)[C@@H]1CC[C@@]2(O)C#C	NIH repository
Benproperine Phosphate	CC(COc1ccccc1Cc2ccccc2)N3CCCCC3.OP(=O)(O)O	NIH repository
Nadolol	CC(C)(C)NCC(O)COc1cccc2C[C@@H](O)[C@@H](O)Cc12	NIH repository
Rifabutin	CO[C@H]1\C=C\O[C@@]2(C)Oc3c(C2=O)c2c(c(O)c3C)c(O)c(NC(=O)\C(C)=C/C=C/[C@H](C)[C@H](O)[C@@H](C)[C@@H](O)[C@@H](C)[C@H](OC(C)=O)[C@@H]1C)c1=NC3(CCN(CC(C)C)CC3)N=c21	NIH repository
Loperamide hydrochloride	CN(C)C(=O)C(CCN1CCC(O)(CC1)c1ccc(Cl)cc1)(c1ccccc1)c1ccccc1.Cl	NIH repository
Brimonidine	Brc1c(NC2=NCCN2)ccc2nccnc12	NIH repository
Ondansetron	Cc1nccn1CC1CCc2c(C1=O)c1ccccc1n2C	NIH repository
Oxaprozin	OC(=O)CCc1nc(c(o1)-c1ccccc1)-c1ccccc1	NIH repository

Diazepam	CN1c2ccc(Cl)cc2C(=NCC1=O)c1ccccc1	NIH repository
Vecuronium Bromide	CC(=O)O[C@H]1[C@H](C[C@H]2[C@@H]3CC[C@H]4C[C@H](OC(C)=O)[C@H](C[C@]4(C)[C@H]3CC[C@]12C)N1CCCCC1)[N+]1(C)CCCCC1.[Br-]	NIH repository
3,5,3' – Triiodothyronine	NC(Cc1cc(I)c(Oc2ccc(O)c(I)c2)c(I)c1)C(O)=O	NIH repository
Amiodarone Hydrochloride	CCCCc1oc2ccccc2c1C(=O)c1cc(I)c(OCCN(CC)CC)c(I)c1.Cl	NIH repository
Deferiprone	Cc1c(O)c(=O)ccn1C	NIH repository
Valproic Acid	CCCC(CCC)C(O)=O	NIH repository
Clofazimine	CC(C)\N=c1/cc2n(-c3ccc(Cl)cc3)c3ccccc3nc2cc1Nc1ccc(Cl)cc1	NIH repository
Raloxifene HCl	Oc1ccc(cc1)-c1sc2cc(O)ccc2c1C(=O)c1ccc(OCCN2CCCCC2)cc1.Cl	NIH repository

4. MD Simulations of Docked Complexes

Figure S3 presents the MD simulation results of the docked complexes for four compounds: 7,8-Dihydroxyflavone, Nadolol, Valproic Acid, and Dicaffeoylquinic Acid. These simulations were performed to examine the stability and potential conformational changes of the protein-ligand complexes over a 10 ns period. The TIP3P model was used for the solvent, and the OPSL4 force field was applied for the simulations. Data were recorded at intervals of 10 ps.

The plots illustrate the root mean square deviation (RMSD) of the protein backbone, heavy atoms, and the ligand over time. Each subfigure (a-d) corresponds to one of the compounds, displaying how the RMSD values fluctuate throughout the simulation, providing insights into the dynamic behavior and interaction stability of each complex.

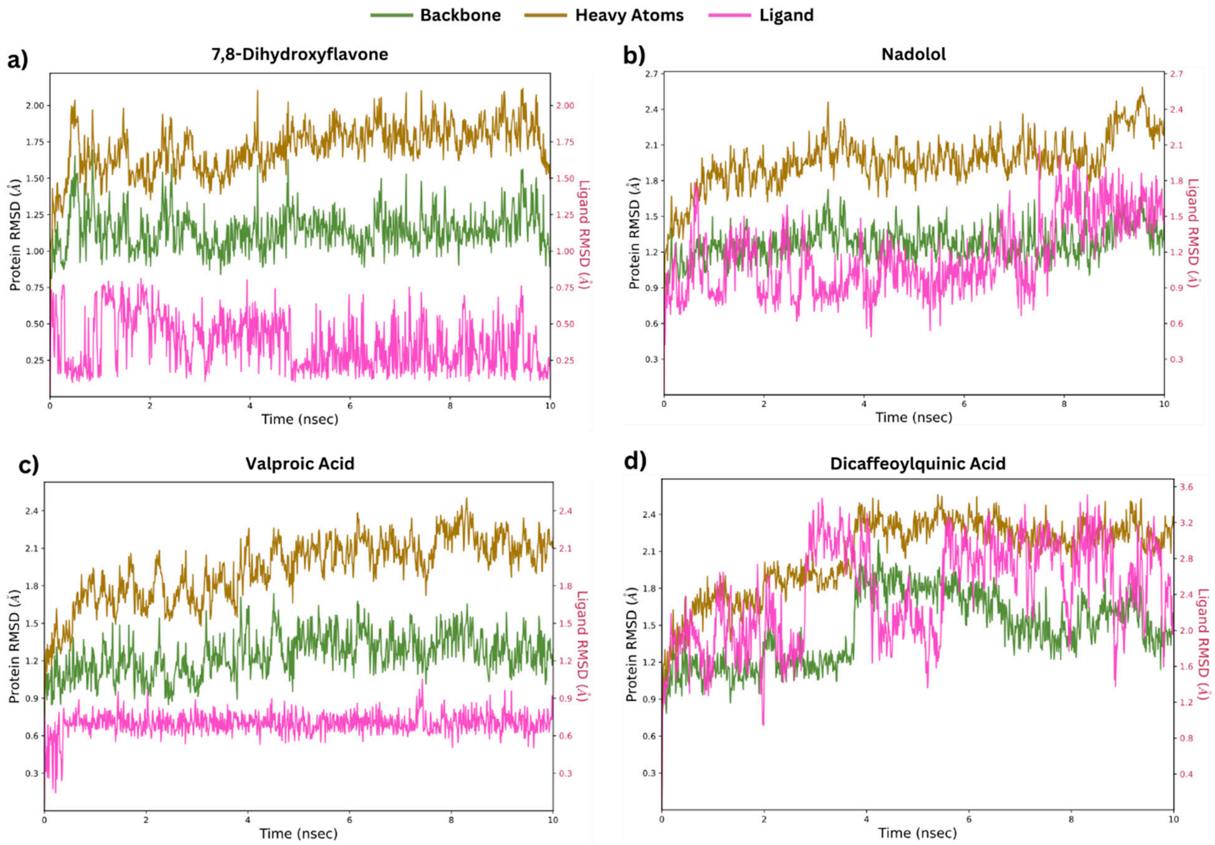

Figure S3. The MD simulations were conducted using the TIP3P model for the solvent and the OPSL4 force field. The total simulation time was 10 ns, with data recorded at intervals of 10 ps.

5. TrkB-D5 Binding Site Analysis

Table S2 presents a detailed analysis of the binding site regions (BP1 to BP5) within the TrkB-D5 protein, highlighting the key amino acids responsible for hydrogen bonding and hydrophobic interactions. Each binding site region is defined by a unique set of amino acids that contribute to the overall stability and function of the protein-ligand complex.

Table S2. Key amino acids involved in forming hydrogen bonds and hydrophobic contacts in each binding site region (BP1-BP5) of the TrkB-D5 protein.

Binding Site Region	Key Amino Acids
BP1	His343, Thr306, Phe305, Gly344, His335, Pro304, Thr296, Asp298, Phe291, Cys345, Val336, Cys302
BP2	Asp298, His299, His300, Trp301, Pro351, Asn350, Thr352, Met379, Ser297, Leu348, Asp349
BP3	Asn355, Ser327, Glu326, Asn325, Pro351, Asp349, Tyr329, Asn350, Ile330, Met354, Tyr319, Leu324, Leu348, Thr352
BP4	Lys312, Pro313, Glu326, Thr332, Ile334, Leu315, Ala314, Cys331, Asn325, Gln316, Leu324, Lys333

BP5	Ser375, Ala376, Gly357, Asp358, Asn356, Asn320, His353, Met354, Trp381, His377, Ile334, Thr332, Leu315, Cys331, Glu326, Asn325, Gln316, Leu324
